# HER3 receptor and its role in the therapeutic management of metastatic breast cancer

**DOI:** 10.1186/s12967-024-05445-8

**Published:** 2024-07-17

**Authors:** Meiying Zhu, Minghui Yu, Yuan Meng, Jie Yang, Xuerui Wang, Longhui LI, Yangyueying Liang, Fanming Kong

**Affiliations:** 1 Department of Oncology, First Teaching Hospital of Tianjin University of Traditional Chinese Medicine, Anshanxi Road, Nankai District, Tianjin, 300193 China; 2 National Clinical Research Center for Chinese Medicine Acupuncture and Moxibustion, Tianjin, China; 3 Tianjin Cancer Institute of Traditional Chinese Medicine, Tianjin, China

**Keywords:** Metastatic breast cancer, HER3, Targeted therapy, Antibody–drug conjugate, Breast cancer

## Abstract

Metastatic breast cancer (mBC) poses a significant threat to women's health and is a major cause of malignant neoplasms in women. Human epidermal growth factor receptor (HER)3, an integral member of the ErbB/HER receptor tyrosine kinase family, is a crucial activator of the phosphoinositide-3 kinase/protein kinase B signaling pathway. HER3 overexpression significantly contributes to the development of resistance to drugs targeting other HER receptors, such as HER2 and epidermal growth factor receptors, and plays a crucial role in the onset and progression of mBC. Recently, numerous HER3-targeted therapeutic agents, such as monoclonal antibodies (mAbs), bispecific antibodies (bAbs), and antibody–drug conjugates (ADCs), have emerged. However, the efficacy of HER3-targeted mAbs and bAbs is limited when used individually, and their combination may result in toxic adverse effects. On the other hand, ADCs are cytotoxic to cancer cells and can bind to target cells through antibodies, which highlights their use in targeted HER3 therapy for mBC. This review provides an overview of recent advancements in HER3 research, historical initiatives, and innovative approaches in targeted HER3 therapy for metastatic breast cancer. Evaluating the advantages and disadvantages of current methods may yield valuable insights and lessons.

## Introduction

**Fig. 1:**
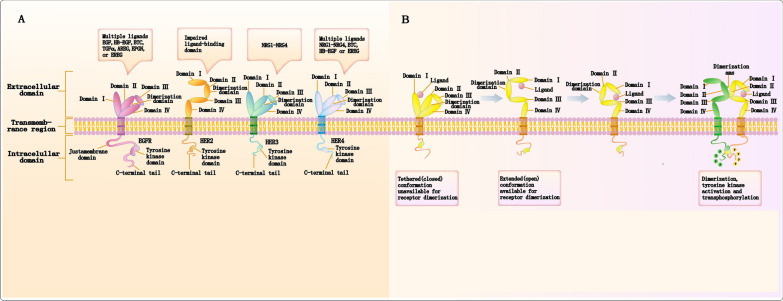
Structure of ErbB receptors, ligands, and conformational changes upon ligand binding. **A** The ErbB family (ErbB1-ErbB4; also referred to as EGFR, HER2, HER3, and HER4) comprises receptor tyrosine kinases (RTKs) with similar structures, each consisting of an extracellular domain (ECD), a single hydrophobic transmembrane region, and an intracellular domain. The intracellular domain includes a juxtamembrane region, a tyrosine kinase domain, and a tyrosine-rich carboxy-terminal tail. The extracellular domain is divided into four subdomains, designated as Subdomains I-IV. EGFR, HER3, and HER4 exist in a tethered ("closed") conformation in the absence of ligands, preventing the dimerization domain from interacting with the corresponding Erbb components. HER2, lacking a known ligand, exists in an active extended ("open") conformation and can readily dimerize permanently. Among EGFR ligands, EGF, transforming growth factor-alpha (TGFα), amphiregulin (AREG), and epigen (EPGN) interact exclusively with EGFR, whereas epiregulin (EREG), heparin-binding EGF-like growth factor (HB-EGF), and betacellulin (BTC) can also bind and activate HER4. A family of EGF-related ligands, the neuregulins (NRGs; composed of NRG1-NRG4), binds to HER3 and HER4. HER2 does not directly bind these EGF-related ligands. HER3 has an impaired tyrosine kinase domain and exhibits reduced kinase activity. Therefore, to activate and facilitate signaling through HER2 and HER3, heterodimerization with other ErbB family members is required. **B** Ligand binding to ErbB receptors induces a conformational change in the molecular fold of the dimerization domain, a step necessary for dimer formation and the functional activation of EGFR, ERBB3, and ERBB4. The interaction in the kinase domains is asymmetrical, where the amino-terminal lobe of one tyrosine kinase interacts with the carboxy-terminal lobe of another

The ErbB family (ErbB1–ErbB4; also known as epidermal growth factor receptor [EGFR], human EGFR [HER]2, HER3, and HER4) comprises receptor tyrosine kinases (RTKs) [1]. Through signal transduction, the ErbB family supports cellular activities on which cell survival and function depend [2]. Each receptor comprises an extracellular ligand–binding domain, a single hydrophobic transmembrane region, and an intracellular segment with a tyrosine kinase domain [3]. EGFR, HER3, and HER4 ligands are currently well-studied [4] (Fig. 1). The downstream signaling pathways activated by ErbB family members are interconnected and overlapping [5–8]. The two main representative signaling pathways are the phosphatidylinositol-3 kinase (PI3K)-protein kinase B (AKT)-mammalian target of rapamycin (mTOR) and mitogen-activated protein kinase (MAPK) pathways [5, 6, 9, 10]. Additionally, there is the phospholipase Cγ–protein kinase C [11, 12], and Janus kinase (JAK)2-signal transducer, and activator of transcription 3 pathways [13, 14]. Aberrant activation of EGFR and HER2 in cancer cells can be induced by numerous mechanisms, including gene amplification, point mutations, deletions, and autocrine ligand-receptor stimulations [8, 15–18]. Alterations in these genes lead to the abnormal activation of EGFR and HER2 signaling, independent of ligand-receptor stimulation, resulting in tumorigenesis, growth, and progression. HER2, located on chromosome 17q12.1, was first identified as a novel gene from rat neuroblastomas that transformed NIH 3T3 cells [19]. King et al. reported that DNA from human breast carcinoma amplifies this gene [20]. HER2 encodes a 185-kDa transmembrane protein [21] that phosphorylates tyrosine residues in the protein kinase domain within the cell through dimerization, activating downstream oncogenic signals [22] and leading to aggressive tumor growth. HER2 amplification is the primary mechanism of HER2 receptor overexpression. The overexpression/amplification of HER2 in a variety of tumors is the main driver of the occurrence and progression of some breast cancers. Of note, HER2 protein overexpression may also be found in the absence of gene amplification [23]. In addition to amplification and overexpression, HER2 increases kinase activity through missense mutations and in-frame insertions, contributing to tumorigenesis [24–27]. In addition, HER2 gene fusion is also a potential therapeutic target [28]. For example, NOS-HER2 and ZNF207-HER2 fusions have been characterized and found to undergo autophosphorylation and cell transformation [29]. Owing to its limited kinase activity [30–33], the oncogenic function of HER3 is mainly mediated through its overexpression and interaction with EGFR or HER2. The role of HER4 in tumorigenesis and progression is inconsistent because it has multiple isoforms, such as oncogenic and tumor-suppressor isoforms, each with different activities [34, 35]. HER3 is overexpressed in various cancers (e.g., breast, colorectal, bladder, melanoma, lung) [36]. Breast cancer (BC) is a prevalent cancer, with 2.3 million new cases and 680,000 deaths reported in the year 2020 [37]. Metastasis, the process by which cancer cells spread, is responsible for mortality associated with breast cancer. Furthermore, a significant proportion of initial-stage cases, ranging from 20 to 30%, advance, resulting in poor survival rates in metastatic BC [38]. HER3 expression is high in mBC. Primary tumors have 30% HER3 expression, which increases to 60% in mBC [39]. HER3 is overexpressed in various cancers, including breast, ovarian, colon, and gastric [36, 40]. However, compared to the broad representation of EGFR mutations, HER3 mutations are rare [41]. Although HER3 itself does not cause tumorigenesis, the HER2:HER3 heterodimer has the highest transforming capacity among all possible EGFR family dimers, and the superior oncogenic capacity of the dimer makes HER3 critical for HER2-mediated tumorigenesis in breast cancer [42, 43]. In addition, inhibition of HER3 expression reversed HER2-dependent tumorigenesis in a transgenic breast tumor model [44]. In breast cancer cell lines, HER3 expression was shown to be essential for maintaining cell viability, whereas EGFR was dispensable. In addition, compared with the broad representation of EGFR mutation, the data rarely HER3 mutations. In breast cancer, several HER3 mutations (F94L, G284R, D297Y, T355I, and E1261A) have been shown to have functional properties [45]. Cote et al. describe three germ line ErbB3 single nucleotide polymorphisms (SNPs), these SNPs cause dorsey his racing, carboplatin chemotherapy drugs and by ErbB2 positive breast cancer patients treated bead sheet resistance disease-free survival rate is poor [46]. At present, most studies use the combination of cytoplasmic and membrane HER3 staining to determine HER3 expression. However, a pooled analysis of studies assessing HER3 in the cytoplasm or membranes did not show a significant difference. To address this knowledge gap, a consistent and reproducible method to evaluate HER3 is warranted to help better identify patients who are more responsive to the anti-HER3 therapies that are currently being evaluated clinically [47].

**Fig. 2:**
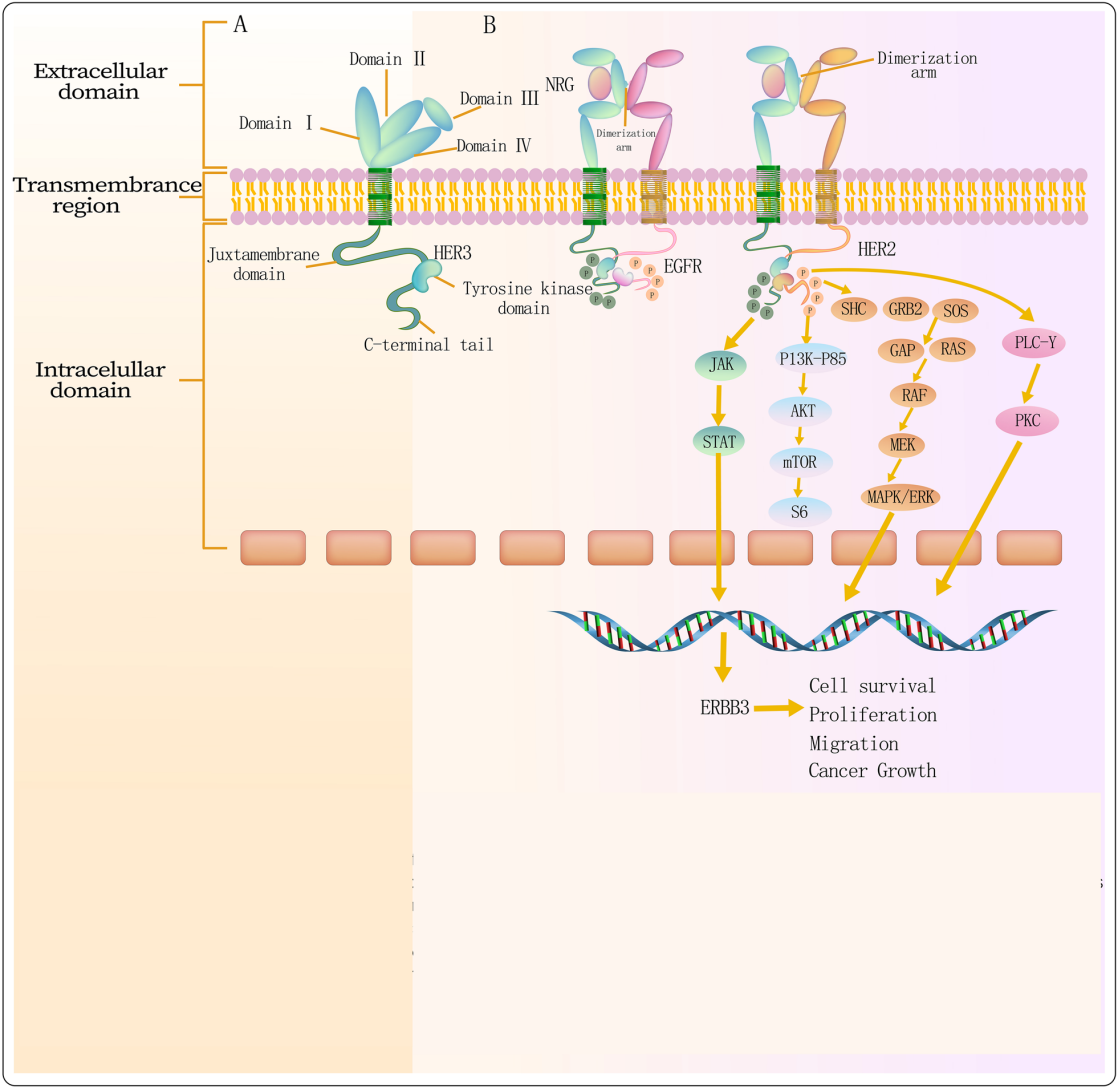
HER3 receptor and its signaling cascade. **A** The monomeric inactive form of HER3 comprises the extracellular, transmembrane, and intracellular regions. The extracellular region comprises subdomains I-IV, with subdomains I and III responsible for ligand binding and subdomain II containing a dimerization arm. The intracellular region is composed of the juxtamembrane domain, tyrosine kinase domain, and C-terminal tail with phosphorylation sites. **B** Upon NRG binding to subdomain I and III, a conformational change occurs in the extracellular region, exposing the dimerization arm. This leads to heterodimerization between HER3 and EGFR/HER2 receptors, subsequently resulting in the phosphorylation of HER3 C-terminal tail and the activation of downstream intracellular signaling cascades, including PI3K/AKT, MAPK, JAK/STAT, SRC, and PLCγ/PKC. These signaling pathways collectively promote cell survival, proliferation, migration, and growth. The abbreviations used are as follows: AKT (protein kinase B), GDP (guanosine diphosphate), GRB2 (growth factor receptor-bound protein 2), JAK (Janus kinase), MAPK (mitogen-activated protein kinase), MEK (mitogen-activated extracellular signal-regulated kinase), SOS (son of sevenless), STAT (signal transducer and activator of transcription)

HER3 is a 180-kDa protein derived from the *ERBB3* gene on chromosome 12q13 [1]. It consists of an intracellular domain containing a juxtamembrane region, a tyrosine kinase domain, a tyrosine-rich carboxy-terminal tail, an extracellular domain (ECD), and a transmembrane domain. The ECD contains four subdomains labeled I through IV, with subdomains II and IV being rich in cysteines. Subdomain II holds a crucial dimerization arm for the interaction of HER3 with other receptors [48] (Fig. 2). Unlike other ErbB family members, HER3's ligands include neuregulins (NRGs), specifically NRG-1 and NRG-2, which are subsets of EGF ligands [49]. Intramolecular interactions keep subdomains II and IV in an inactive conformation without ligands. Ligand binding alters the ECD structure, resulting in an open conformation [50] and exposing the dimerization arm in subdomain II, allowing HER3 to form heterodimers with ErbB RTK monomers. The kinase domains engage asymmetrically, leading to transphosphorylation [22] (Fig. 2). Reports indicate that HER2 and HER3 pairing requires NRGs [51]. These heterodimers have potent transforming potential, essential for HER2-driven tumorigenesis. HER3 overexpression alone lacks the carcinogenic effect seen in other ErbB members [41]. It can also heterodimerize with non-ErbB receptors such as MET and FGFR2 [52, 53]. The kinase domain of HER3 has an inactive conformation of a key tyrosine residue, which results in approximately 1,000 times lower tyrosine kinase activity compared to EGFR [54]. However, HER2 (and/or EGFR) heterodimers amplify signaling, providing a potent stimulus for human breast cancer [49]. HER3 can bind to the p85 subunit of PI3K, triggering the activation of PI3K/AKT signaling, which is crucial for tumor cell survival [55]. Additionally, HER3 activates pathways, including the MAPK cascade, JAK, and the oncogene *c-Src (SRC)* [56] (Fig. 2).

Increased HER3 expression reduces the effectiveness of EGFR-targeted treatment by causing non-EGFR heterodimer formation, leading to EGFR-targeted tyrosine kinase inhibitor (TKI) resistance [55]. Studies have shown that hepatocyte growth factor receptor (MET) amplification increases gefitinib resistance by increasing HER3/PI3K signaling. MET amplification was detected in 22% of patients with lung cancer resistant to gefitinib or erlotinib [52]. In addition, HER3 signaling is associated with resistance to the EGFR-targeted TKIs gefitinib in head and neck squamous cell carcinoma (HNSCC) and BC [57, 58]. Lapatinib, a dual TKI targeting EGFR and HER2, induces feedback upregulation at the mRNA and protein levels in BC cell lines, and HER3 knockdown restores the drug sensitivity of lapatinib-resistant cells [59]. Lapatinib-resistant cells in BC cell lines depend on the heregulin (HRG)-driven HER3-EGFR-PI3K-PDK1 signaling axis [60]. Another study showed that lapatinib could induce symmetric HER2:HER3 dimers, which may promote tumor cell proliferation [61] (Table 1). In EGFR–TKI–treated BC, HER3 is related to gefitinib resistance [58], whereas cetuximab/panitumumab-resistant patients with BC show increased EGFR: HER3 heterodimerization [62]. Patritumab effectively counteracts the resistance to EGFR inhibitors caused by NRG1, with the levels of circulating NRG1 being a more reliable indicator of its efficiency compared to the expression of HER3 mRNA [63]. HER3 overexpression hampers HER2-targeted therapies, as observed in trastuzumab resistance in HER2-positive mBC [63]. NRG1 stimulation induces HER3 overexpression, triggering PI3K/AKT and SRC pathways and HER2–IGF–1R heterotrimer formation, underpinning trastuzumab resistance [64]. Trastuzumab sensitivity is restored by HER3 expression reduction, while hormone and chemotherapy resistance is conferred by high HER3 expression. In HER2-positive BC, HER3 overexpression results in tamoxifen resistance [65] and is associated with resistance to fulvestrant [66] and paclitaxel, whereas inhibiting HER3 overexpression restores drug sensitivity [67]. Targeted HER3 therapies involving inhibition of HER3 kinase activity, blocking heterodimerization, and using monoclonal antibodies such as seribantumab, lumretuzumab, elgemtumab, and patritumab are being developed to treat mBC. However, the effectiveness of single treatments is limited. Combining multiple therapies, such as elgemtumab, trastuzumab, and alpelisib, may lead to harmful adverse effects [67]. However, monoclonal/dual-specificity antibodies have modest mBC efficacy alone or when used in combination. Consequently, the targeted distribution of the cytotoxic payload has been achieved by using antibody–drug conjugates (ADCs). With its ability to cause immunological stimulation, antitumor response, and cell damage, patritumab deruxtecan (U3-1402) shows promise as a treatment for late-stage breast cancer. ADCs present a viable way to improve mBC care and increase survival. Overcoming resistance, lowering toxicity, and increasing tumor cell absorption are among the difficulties. Finding predictive biomarkers is therefore essential when selecting patients.

**Table 1:** A summary of HER3 overexpression and drug resistance mechanisms

Drug type	Drug name	Targets	Conclusion	References
Tyrosine kinase inhibitors	Gefitinib	EGFR	In breast cancer cell lines, the signaling mediated by HER2:HER3 is associated with resistance to gefitinib, as HER3 and the PI(3)K/Akt pathway are not effectively inhibited by gefitinib	[58]
	Lapatinib	EGFR HER2	In breast cancer cell lines, lapatinib-resistant cells rely on the HER3-EGFR-PI3K-PDK1 signaling axis driven by heregulin (HRG)	[60]
			Studies indicate that treatment with lapatinib can induce a feedback upregulation of mRNA and protein levels in breast cancer cell lines, and knockdown of HER3 can restore drug sensitivity in lapatinib-resistant cells	[59]
			Research demonstrates that lapatinib can induce symmetric HER2:HER3 dimers, which may promote tumor cell proliferation	[61]
The blocking Abs	Trastuzumab	HER2	Two studies suggest that in breast cancer cell lines, bypass activation of PI3K/AKT and SRC driven by HER3 is a primary mechanism of therapy resistance	[64, 145]
			Another study indicates that the formation of heterotrimeric complexes of HER2, HER3, and IGF1R is a principal inducer of trastuzumab resistance driven by AKT and SRC in breast cancer cells	[56]
Hormonal therapy	Tamoxifen	ER	HER3 is critical in the phosphorylation of HER2 in breast cancer cells, and overexpression of HER3 can lead to tamoxifen resistance	[146]
			Studies indicate that patients with breast cancer co-expressing HER2 and HER3 are more likely to develop tamoxifen resistance	[147, 148]
	Fulvestrant	ER	Increased activity of EGFR, HER2, and HER3 is associated with resistance to the ER agonist fulvestrant	[66]
			Treatment with fulvestrant promotes the expression and phosphorylation of HER3 in breast cancer cells, underlying the mechanism of resistance to fulvestrant in breast cancer	[149]
Chemotherapy	Paclitaxel	–	In HER2-positive breast cancer cell lines, resistance to paclitaxel is associated with upregulation of HER3 and increased levels of Survivin	[67]
Chemotherapy	Paclitaxel Doxorubicin 5-fluorouracil Etoposide Camptothecin	–	In breast cancer cell lines, resistance to various chemotherapeutic agents such as 5-fluorouracil, paclitaxel, camptothecin, and etoposide is associated with co-expression of HER2/HER3 and the PI3K/AKT signaling pathway	[150]

## Targeting HER3 to treat mBC

**Fig. 3:**
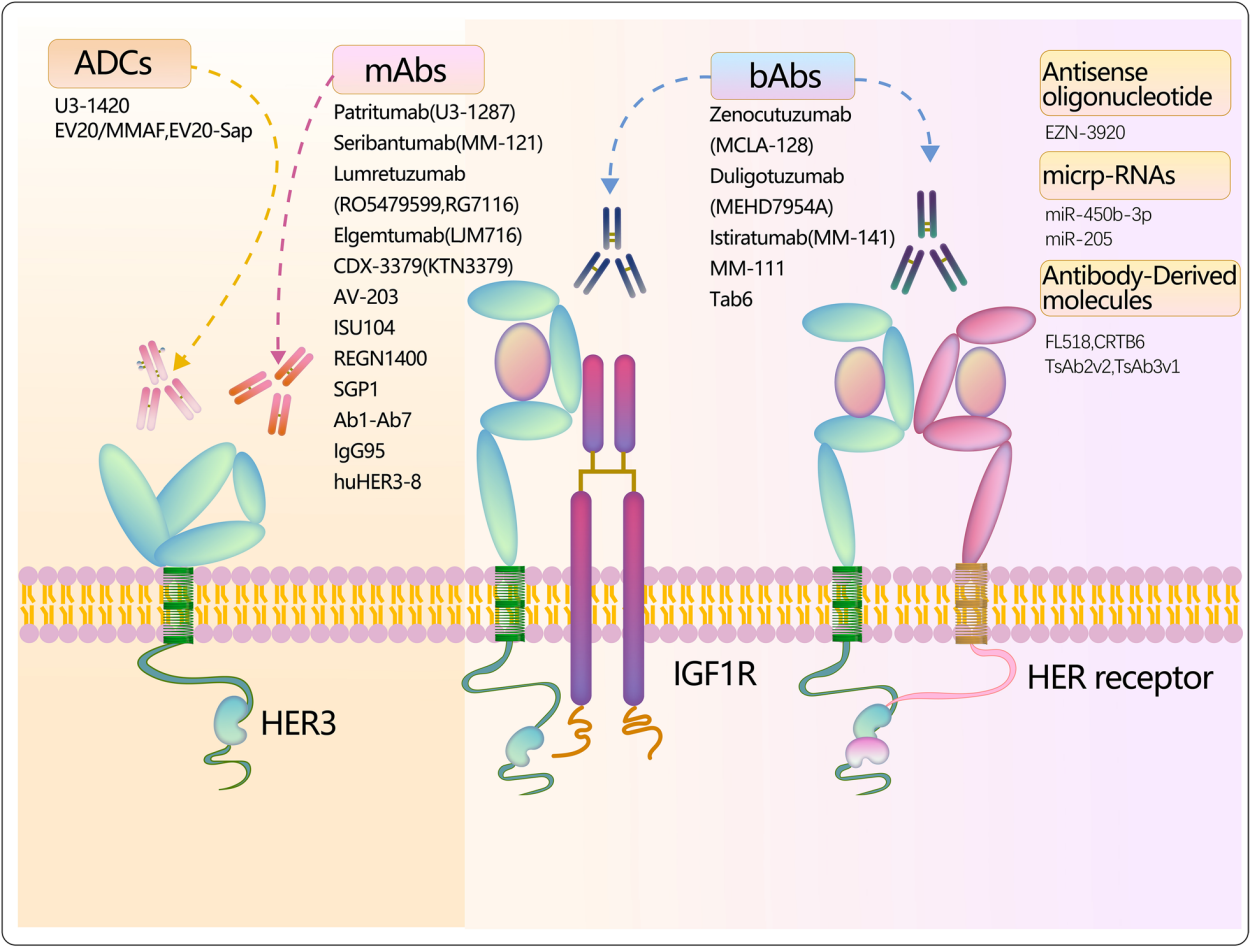
The current status quo of HER3-targeted therapies for MBC. Monoclonals antibodies (mAbs), bispecific antibodies (bAbs), antibody–drug conjugate (ADC) and other therapies such as antibody-derived molecules

The prevailing strategies for HER3-targeted therapy in mBC involve the use of antibodies targeting the extracellular domain of HER3, as detailed in the following sections. Figure 3 and Table 2 briefly outline the current status of drugs in preclinical and clinical trials for HER3-targeted therapy in mBC.

**Table 2:** A summary of the HER3-targeted therapies for MBC under evaluation in preclinical and clinical trials

Drug type	Drug name	Mechanism of action	Developer	Highest clinical trial phase	Key trial numbers	References
Antibodies	Lumretuzumab (R05479599, RG7116)	HER3 mAb	Genentech/Roche	PhWase Ib/II	NCT01918254 NCT02204345 NCT01482377	[69, 70]
	Seribantumab (MM-121)	HER3 mAb	Merrimack Pharmaceuticals/ Elevation oncology	Phase II	NCT00994123	
					NCT01209195	[71–73]
					NCT01451632	
					NCT04383210	
					NCT02134015	
	AMG-888, (U3-1287)	HER3 mAb	Daiichi sankyo	Phase III	NCT02134015	[74, 75, 85]
					NCT01211483	
					NCT02143622	
	Elgemtumab (LJM716)	HER3 mAb	Morphosys/novartis	Phase I/II	NCT01602406	[76–78]
					NCT02167854	
	ISU104	HER3 mAb	ISU Abxis	Phase I	NCT03552406	[79]
	CDX-3379 (KTN3379)	HER3 mAb	Celldex therapeutics	Phase II	NCT02473731 NCT02014909	[80]
	AV-203	HER3 mAb	Aveo oncology	Phase I	NCT01603979	[81, 82]
	REGN1400	HER3 mAb	Regeneron Pharmaceutials	Phase I	NCT01727869	[83, 84]
	1F9E5	HER3 mAb	–	Preclinical	–	[86]
	TAMHER3	HER3 mAb	–	Preclinical	–	[87]
	SGP1	HER3 mAb	–	Preclinical	–	[88–90]
	Ab1-Ab7	HER3 mAb	–	Preclinical	–	[91]
	IgG95	HER3mAb	–	Preclinical	–	[92]
	huHER3-8	HER3 mAb	–	Preclinical	–	[93]
ADCs	U3-1402	HER3-ADC	Daiichi sankyo	Phase II	NCT04479436 NCT03260491 NCT02980341	[78, 113, 114]
	EVA20/MMAF	HER3-ADC	Mediapharma	Preclinical	–	[115]
	EV20-Sap	HER3-ADC	Mediapharma	Preclinical	–	[115]
Bi-specific antibodies	Zenocutuzumab (MCLA-128)	HER2/HER3 bispecific	Merus	Phase I/II	NCT02912949	[94, 95]
	MM-111	HER2/HER3 bispecific	Merrimack Pharmaceuticals	Phase I	NCT01097460 NCT00911898 NCT01304784	[96, 97]
	Istiratumab(MM-141)	HER3/IGF1R bispecific	Merrimack Pharmaceuticals	Phase II	NCT01733004 NCT02538627 NCT02399137	[98]
	Duligotuzumab (MEHD7954A)	EGFR/HER3 bispecific	Genetech/Roche	Phase II	NCT01911598 NCT01986166	[62, 99–107]
	Tab6	HER2/HER3 bispecific	–	Preclinical	–	[108]
Antisense olihonucleotide	EZN-3920	HER3 mRNA antagonist	Enzon Pharmaceuticals	Preclinical	–	[118]
micro-RNAs	miR-450b-3p	Inhibits HER3 expression	–	Preclinical	–	[119–121]
	miR-205	Inhibits HER3 expression	–	Preclinical	–	
Antibody-derived molecules	FL518, CRTB6	HER3/EGFR bAb		Preclinical		[116]
		HER2/VEFR bAb	–		–	
		mixture				
Antibody-derived molecules	TsAb2v2, TsAb3v1	RGFR/HER3/cMet/IGFIR	–	Preclinical	–	[117]
		mAb mixture				
HER3 vaccine	Ad-HER3,Ad-HER3-FL	HER3 vaccine	–	Preclinical	–	[128–130]

### Monoclonal antibodies

Monoclonal antibodies (MAbs) targeting HER3 have been engineered to disrupt receptor-ligand binding or hinder heterodimerization [68]. Preclinical and clinical studies have been conducted on several mAbs targeting HER3, some of which have advanced to phase 1 trials to evaluate their safety, tolerability, and preliminary efficacy in solid tumors. Some mAbs have progressed to phase 2 and 3 trials, such as seribantumab, lumretuzumab, and patritumab.

Lumretuzumab (RG7116, RO5479599 GE-huMab-HER3) is a humanized glycoengineered immunoglobulin (Ig) G1 targeting subdomain I of the HER3 ECD [69]. This antibody prevents the attachment of NRG to the HER3 receptor, thereby impeding the formation of receptor heterodimers and activating the immune system to induce lethal effects. Lumretuzumab further reduces HER3 expression. In a xenograft model of ER + /HER3 + /HER2–low human BC, the combination of lumretuzumab and pertuzumab demonstrated efficacy, leading to tumor regression [70].

Seribantumab (MM-121, SAR256212) is a human IgG2 mAb that competes with NRG for HER3 binding, obstructing dimerization and inhibiting HRG-mediated downstream PI3K/AKT signaling [71]. Seribantumab also induces HER3 internalization and degradation. In combination with trastuzumab, seribantumab inhibits the growth of HER2 + mBC cells. MM-121 also exhibits enhanced antitumor activity in HER2 + mBC frameworks resistant to paclitaxel and trastuzumab [72]. The phase 2 investigation of seribantumab and exemestane in hormone receptor-positive HER2-negative patients with mBC highlights the therapeutic advantages of the combination in the HRG-high subgroup in the context of biomarker assessment [73].

AMG-888, also known as U3-1287, is a human IgG1 mAb that completely inhibits ligand binding to HER3, causing receptor internalization and destruction. Patients with HER2-positive mBC may have a prolonged overall survival with patritumab [74]. A phase 1 study has reported that the combination of patritumab with paclitaxel and trastuzumab in patients with HER2-positive mBC exhibited controllable toxicity and promising initial activity [75].

Elgemtumab (LJM716) is a human IgG1 mAb that binds to an epitope between the HER3 ECD subdomains II and IV, leading to conformation closure and blocking receptor activation [76]. Elgemtumab combined with trastuzumab and lapatinib enhanced the survival of HER2-positive BC xenograft mice [77]. Elgemtumab, however, showed significant gastrointestinal toxicity when combined with trastuzumab and alpelisib in patients with PI3K-mutant HER2-positive mBC, indicating that combination therapy targeting this pathway should be used with caution [78].

ISU104 is an antibody that targets HER3 and binds to subdomain III. ISU104 suppresses HER3, prevents receptor attachment to NRG, inhibits receptor heterodimerization with other HER receptors, and inactivates downstream signaling. In xenograft BC models, ISU104 showed more than 70% suppression of tumor development [79].

CDX-3379 (KTN3379) is a human mAb (IgG1λ) that binds to an exclusive epitope between subdomains II and III with high affinity, stabilizing HER3 in an inactive state. Therefore, the antibody inhibits both ligand-dependent and ligand-independent HER3 activation. CDX-3379 shows therapeutic effects in HER2-positive BC xenograft tumor models [80].

AV-203 (CAN017) is a human IgG1 mAb that inhibits the binding of HER3 to NRG. It has demonstrated tumor growth inhibition in breast cancer (BC) xenograft models [81]. It was discovered to inhibit tumor growth in BC xenograft frameworks. A phase 1 clinical trial reported AV-203 to be safe for use in patients with metastatic breast cancer (mBC) or advanced BC [82].

REGN1400, a fully human IgG mAb, effectively blocks the interaction between HER3 and NRG, hindering the proliferation of BC cells. REGN1400 shows promising efficacy in inhibiting tumor growth when administered in combination with EGFR or HER2-targeted antibodies [83]. Phase I trials studying the combination of REGN1400 with erlotinib or cetuximab have reported satisfactory tolerability [84].

Several drugs show inhibitory effects on the growth and proliferation of tumor cells in preclinical studies. For example, the first fully humanized HER3 mAb U3-1287 can inhibit tumor growth in a BC xenotransplantation model [85]. The anti-HER3 mAb 1F9E5 has shown similar inhibitory effects on cell proliferation to trastuzumab and is more effective than other anti-HER3 monoclonal antibodies alone or in combination with trastuzumab [86]. The TAMHER3 construct created by Anna Orlova et al. can inhibit the growth of HER3-expressing xenograft tumors and shows good tolerance in mice without adverse events or weight loss [87].

SGP1 is an antibody that targets HER3 and competes with NRG for binding to the HER3 receptor [88]. This antibody effectively inhibits NRG-induced tumor cell growth and shows an enhanced growth-inhibiting effect in mBC cells when combined with trastuzumab [89]. SGP1, as monotherapy or in combination with lapatinib, inhibits the proliferation of lapatinib-resistant HER2-positive mBC cells [90].

Okita et al. produced rat mAbs (Ab1–Ab7) targeting HER3, which could induce HER3 internalization and inhibit NRG binding, HER3 phosphorylation, and cell growth. The combination of Ab4 and erlotinib shows promising therapeutic effects in treating HER2-positive mBC [91].

Turowec et al. developed IgG95, which blocks the binding of ligands to the HER3 receptor, leading to the downregulation of HER3 expression. This antibody inhibits the proliferation of HER2-amplified mBC cells [92].

The mouse anti-HER3 antibody, HER3-8, demonstrates efficacy in inhibiting HER2:HER3 dimerization and decreasing the proliferation of BC cell lines stimulated by ligands. Accordingly, efforts have been made to humanize the HER3-8 antibody, and the product has been designated as huHER3-8 [93].

### Bispecific antibodies

In clinical studies, bivalent antibodies (BAbs) targeting two antigens simultaneously have been investigated to address the limitations and resistance mechanisms of single-agent HER3-targeted mAbs. Zenocutuzumab (MCLA-128) is an IgG1 bAb that targets both HER2 (subdomain I) and HER3 (subdomain III). Zenocutuzumab docks onto HER2, preventing ligands from binding to HER3 and disrupting HER2-HER3 heterodimers, thereby inhibiting oncogenic signaling. This bAb has demonstrated efficacy in NRG1 fusion-positive mBC that is resistant to chemotherapy [94]. Zenocutuzumab is currently in phase 1/2 clinical trials, showing favorable tolerability, safety, and anti-cancer efficacy [95].

MM-111 is a bAb targeting both HER2 and HER3. Its anti-HER2 arm positions the bAb within HER2 + tumor cells, while its anti-HER3 arm blocks NRG binding with HER3 [96]. Preclinical research has demonstrated that MM-111, in combination with trastuzumab or lapatinib, increases anti-cancer activity in HER2-positive mBC cells [97]. A clinical investigation found that the combination of MM-111 with standard HER2-targeted treatment and chemotherapy was safe [96].

Istiratumab (MM-141) targets both HER3 and IGF-1R. It entered phase 2 trials for pancreatic cancer [98] but has not yet entered clinical trials for BC.

Duligotuzumab (MEDH7945A) is a humanized IgG1 bAb targeting EGFR and HER3. It binds to the extracellular domains of EGFR or HER3, blocks ligand binding, inhibits signaling pathways, and enhances antibody-dependent cell-mediated cytotoxicity [99, 100]. Several clinical trials (phase 1/2) are evaluating duligotuzumab and have generally reported limited activity [101–105]. In preclinical trials, duligotuzumab has shown efficacy in erlotinib- and cetuximab-resistance models in HNSCC and non-small cell lung cancer [106] or when used in combination with cisplatin [107]. It has also shown efficacy in combination with AKT and PI3K inhibitors in triple-negative breast cancer (TNBC) [62].

TAb6, also known as TA, is a bispecific antibody explicitly targeting both HER2 and HER3. It is produced by combining the anti-HER2 antibody trastuzumab with HER3-specific single-chain fragment variations [108]. However, treatment with TAb6 increases the proliferation of HER2-positive mBC cell lines [108].

### Antibody–drug conjugates

ADCs are a novel class of anti-cancer drugs with significant potential in HER3-targeted therapy. ADCs are composed of targeted mAbs that are chemically linked to cytotoxic drugs, either cleavable or non-cleavable [109]. The mAb component of ADCs binds to specific antigens on the surface of cancer cells, resulting in the internalization of ADCs [110]. ADCs are intended to be transported to lysosomes after internalization, where they release the cytotoxic drug in a proteolytic or acidic environment to cause cell death. ADCs not only specifically target cancer cells but also cause the bystander effect, which is the elimination of surrounding cells regardless of how much the target antigen is expressed. This effect may improve the effectiveness of ADCs, but concerns regarding potential harm to normal cells still exist [111].

ADCs integrate the selective targeting of antibodies with the cytotoxicity of chemotherapeutic drugs, making them a promising class of anti-cancer agents [112]. Currently, three ADCs targeting HER2 have been approved, with others targeting the HER family of receptors under investigation.

U3-1402 (patritumab deruxtecan, HER3-DXd) is an ADC involving the covalent binding of patritumab to a therapeutic linker containing deruxtecan (DX-8951, a topoisomerase I inhibitor). This ADC efficiently prevents DNA replication, which triggers apoptosis. Moreover, U3-1402 may induce cellular damage and immune activation, thereby triggering antitumor immune responses [113]. U3-1402 has demonstrated efficacy in late-stage, heavily pretreated mBC [78]. In a phase 1/2 study, the overall response rate (ORR) was 30.1% for hormone receptor-positive (HR +)/HER2-negative BC (n = 113), 22.6% for TNBC (n = 53), and 42.9% for HER2-positive disease (n = 14). In the SOLTI-TOT HER3 window-of-opportunity trial, a single dose of U3-1402 was evaluated as neoadjuvant therapy in HR + /HER2-negative BC, wherein it demonstrated an ORR of 45%, increased tumor cell activity, and cancer-infiltrating lymphocyte scoring with no significant correlation between response and pretreatment HER3 mRNA levels [114]. U3-1402 was assessed in 182 extensively pretreated patients with HER3-expressing mBC in the U31402-A-J101 phase 1/2 trial, showing prolonged anti-cancer efficacy across all BC subtypes.

Gianluca Sala et al. developed four ADC versions from the anti-HER3 antibody EV20: (1) EV20-sap, conjugated with the plant-derived toxin saporin, (2) EV20/MMAF, (3) EV20-ss-vc/MMAF, conjugated with cleavable or non-cleavable linkers and the cytotoxic drug auristatin F, and (4) EV20/NMS-P945, conjugated with a connectable cleavable linker and a small molecule DNA alkylating agent (thieno indole NMS-P528). Among these, EV20/MMAF demonstrated HER3-dependent cell-killing activity in HER2-positive mBC cell lines [115].

### Antibody-derived molecules

Hu et al. produced tetraspecific antibodies, FL518 and CRTB6, capable of recognizing EGFR, HER2, HER3, and VEGF. CRTB6 was prepared by fusing the variable regions of cetuximab, trastuzumab, lumretuzumab, and bevacizumab into a DVD-Ig-like antibody along with FL518. The combination of duligotuzumab, which targets HER3 and EGFR, and bH1-44, which targets HER2 and VEGF, allowed the tetraspecific antibodies to more effectively block the proliferation and signaling of mBC cells than the bAbs [116].

TsAb2v2 and TsAb3v1 are tetraspecific, tetravalent, Fc-containing antibodies targeting EGFR, HER3, cMet, and IgF1R, with binding arms derived from imgatuzumab (anti-EGFR mAb), lumretuzumab (anti-HER3 mAb), onartuzumab (anti-cMet mAb), and R1507 (anti-IgF1R mAb). When compared to single monoclonal antibodies, or bAbs, the antibodies show higher levels of mBC cell apoptosis and growth inhibition while concurrently binding and inhibiting all targets [117].

### Other anti-HER3 strategies for mBC therapy

Certain antisense oligonucleotides or microRNAs have demonstrated potential efficacy against mBC by downregulating HER3 and preventing tumor cell proliferation. In vitro and xenograft tumor models have shown that EZN-3920, a HER3 antisense oligonucleotide, exhibits anti-cancer efficacy either alone or in combination with TKIs, including models of HER-targeted therapeutic resistance [118]. Several microRNAs (miRNAs), such as miR-125a, miR-125b, miR-205, and miR-450b-3p, suppress HER3 expression by directly targeting the 3′ UTR of HER3 mRNA, inhibiting breast cancer (BC) cell proliferation and showing potential for treating mBC [119–121]. HER3 siRNAs reduce tumor cell proliferation and sensitize cells to targeted HER therapy. HER3 aptamers, which are engineered single-stranded DNA or RNA oligonucleotides, bind to HER3 and have been used to target HER3-positive tumor cells [122]. Yu et al. reported an antitumor effect in HER2-positive BC using a 3-in-1 nucleic acid aptamer–siRNA chimera [123]. Shu et al. have recently shown that carbon dots/HER3 siRNA, alone or in combination with trastuzumab, can inhibit the proliferation of HER2-positive BC cells [124]. HER3 aptamer–protamine–siRNA (targeting oncogenes or CDKs) exerts anti-cancer effects in HER3-positive BC models [125]. A30, an RNA aptamer that targets HER3-ECD, suppresses BC cell proliferation by blocking NRG signaling [126]. A30 has also been used to deliver a set of cytotoxic siRNAs that can inhibit HER3 + BC cell growth [127].

In this domain, preclinical trials have been conducted to evaluate a vaccine that encodes the full-length human HER3 receptor (Ad-HER3 or Ad-HER3-FL) using an adenovirus. Ad-HER3 can efficiently stimulate anti-HER3 antibody production and T-cell responses against cancers. Even in individuals who have become resistant to HER2-targeted therapy, these antibodies may be helpful against mBC. Additionally, there are reports of increased effectiveness when Ad-HER3-FL is used in combination with PD-1/PD-L1 and CTLA4 dual-blockade therapy, suggesting HER3 as a promising target for antitumor vaccines [128, 129]. Furthermore, a preclinical study found that the HER3 vaccine antibody and HER3 peptide mimetic could inhibit cancer cell proliferation and receptor phosphorylation and induce apoptosis and antibody-dependent cytotoxicity [130].

Chimeric antigen receptor-regulated T lymphocytes (CAR-T) can stimulate anti-cancer immunity. However, insufficient tumor specificity of the targeted antigen often leads to immunotoxicity and off-target effects. Endogenous ligands of tumor antigens may be more suitable than single-chain variable fragments as components of chimeric antigen receptors, with high cancer-recognition potential and minimal immunogenicity [131, 132]. A study has shown that CAR-T cells based on the extracellular domain of HRG1β, a natural ligand of HER3/HER4, can effectively inhibit HER family receptor–driven BC, which may provide a new strategy to overcome tumor resistance to HER2-targeted therapy [133].

## Discussion

Despite the acceptable safety profiles of mAbs and bAbs observed in clinical trials, their efficacy has been underwhelming. Moreover, combination strategies with mAbs and bAbs have been hindered by toxicity [134] or inadequate effectiveness [135], leading to the discontinuation of development efforts for several mAbs and bAbs. Conversely, HER3-targeted ADCs offer a novel approach to cancer treatment and have the potential to be a unique, effective therapeutic option for patients with metastatic breast cancer by lowering drug resistance, enhancing treatment efficacy, and minimizing systemic toxicity.

ADCs are rapidly emerging as targeted drugs, although they continue to face substantial obstacles. Improving ADC absorption by cancer cells is a critical issue in developing ADCs. Furthermore, ADCs rely on high levels of target antigen expression on cancer cell surfaces to effectively internalize and release cytotoxic drugs. The limited expression of target antigens on cancer cell surfaces restricts the effectiveness of ADCs. Consequently, patients with limited antigen expression may benefit from increased ADC uptake by cancer cells. Systemic toxicity is another challenge that leads to ADC failure in clinical trials. Toxicity is associated with multiple factors such as inadequate ADC internalization, nonspecific binding of antibodies to Fc receptors [112], premature cleavage and release of free drugs from the linker [136], the bystander effect induced by the super-cytotoxic payload in normal cells [137], and off-target toxicity due to the low expression of target receptors in normal tissues [138]. Furthermore, one issue that needs to be addressed is resistance to ADC therapy.

Potential avenues for future ADC research include the following strategies: (1) Exploring recombinant antibody approaches to enhance ADC internalization and lysosomal trafficking: Studies related to the domains of bAbs and bispecific affinity molecules to augment ADC internalization and lysosomal trafficking are ongoing [139]. As an alternative to antibodies, antibody recombinants, including lysosome-sorting or cell-penetrating peptides, are being investigated [140]. (2) Study of novel payload platforms, conjugation technologies, and linking strategies to improve ADC efficacy while reducing toxicity: Currently, advancements in this field are driven by next-generation ADCs demonstrating enhanced effectiveness, reduced toxicity, and diverse modes of action [141]. Designing cleavable linkers and explicating their release mechanisms could be a key focus in the future [142]. The development of reliable and site-specific conjugation strategies is ongoing to ensure the production of homogeneous ADCs with consistent quality [143]. (3) Improving ADC therapy through clinical and translational research, the possibility of combination therapy to improve drug efficacy and decrease ADC resistance has been considered [144]. (4) Studying the clinical biomarkers will improve patient selection and help monitor response signals to augment ADC efficacy [144].

An adenovirus-based vaccine known as Ad-HER3 or Ad-HER3-FL, encoding the full-length human HER3 receptor, has been developed and evaluated in preclinical trials. It has been discovered that ad-HER3 effectively stimulates T-cell responses against malignancies and generates antibodies against HER3. Even when mBC patients exhibit resistance to HER2-targeted therapies, these antibodies may be useful against the disease. Additionally, reports of increased effectiveness when Ad-HER3-FL is used alongside PD-1/PD-L1 and CTLA4 dual blockade therapy suggest that HER3 could be a promising target for antitumor vaccines [128, 129].

## Conclusions

Overexpression of HER3 plays a crucial role in promoting mBC and providing resistance to treatments targeting HER receptors and chemotherapeutic agents. Although HER3 was identified more than 30 years ago, no therapeutic interventions have reached clinical approval to date. While mAbs and bAbs have shown some effectiveness, their clinical benefits are limited, leading to the discontinuation of their development. The failure of these antibody therapies may be due to the use of incorrect antibody epitopes, pharmacokinetic problems, or a lack of biomarkers. In addition, targeting ErbB3 in isolation may not be sufficient to fully inhibit cancer cell signaling. Therefore, combination therapy targeting ErbB3 and other anti-ErbB receptors or growth factor receptors, as well as hormonal therapy, chemotherapy, immunotherapy, or radiotherapy, may enhance therapeutic effects. ADCs exhibit potential in cancer treatment and offer a promising therapeutic advantage for managing mBC. Delivering therapeutics to ErbB3-expressing cancer cells with anti-ErbB3 ADCs is a promising area for clinical trials. Moreover, the expression of the receptor alone is necessary but not sufficient for the response to ErbB3 therapies. Emerging data suggest that more sophisticated biomarkers are needed. Therefore, to improve clinical outcomes, prospective biomarker validation and HER3-targeted drug studies are essential.

## Data Availability

Not applicable.

## Abbreviations

ADC: Antibody–drug conjugate
AKT: Protein kinase B
bAb: Bispecific antibody
BC: Breast cancer
ECD: Extracellular domain
EGF: Epidermal growth factor
EGFR: EGF receptor
FGFR2: Fibroblast growth factor receptor 2
HER: Human epidermal growth factor receptor
HNSCC: Head and neck squamous cell carcinoma
HR +: Hormone receptor-positive
HRG: Heregulin
IGF: Insulin-like growth factor
IgG: Immunoglobulin G
JAK: Janus kinase
mAb: Monoclonal antibody
MAPK: Mitogen-activated protein kinase
mBC: Metastatic BC
MET: Hepatocyte growth factor receptor
NRG: Neuregulin
ORR: Overall response rate
PI3K: Phosphoinositide-3 kinase
RTK: Receptor tyrosine kinase
SRC: Oncogene c-Src
TKI: Tyrosine kinase inhibitor
TNBC: Triple-negative BC
